# Porcine FcγRIIb Mediates Enhancement of Porcine Reproductive and Respiratory Syndrome Virus (PRRSV) Infection

**DOI:** 10.1371/journal.pone.0028721

**Published:** 2011-12-29

**Authors:** Songlin Qiao, Zhizheng Jiang, Xiaohui Tian, Rui Wang, Guangxu Xing, Bo Wan, Dengke Bao, Yonghui Liu, Huifang Hao, Junqing Guo, Gaiping Zhang

**Affiliations:** Key Laboratory of Animal Immunology of the Ministry of Agriculture, Henan Provincial Key Laboratory of Animal Immunology, Henan Academy of Agricultural Sciences, Zhengzhou, China; University of Georgia, United States of America

## Abstract

Antibody-dependent enhancement (ADE) of virus infection caused by the uptake of virus-antibody complexes by FcγRs is a significant obstacle to the development of effective vaccines to control certain human and animal viral diseases. The activation FcγRs, including FcγRI and FcγRIIa have been shown to mediate ADE infection of virus. In the present paper, we showed that pocine FcγRIIb, an inhibitory FcγR, mediates ADE of PRRSV infection. Stable Marc-145 cell lines expressing poFcγRIIb (Marc-poFcγRII) were established. The relative yield of progeny virus was significantly increased in the presence of sub-neutralization anti-PRRSV antibody. The Fab fragment and normal porcine sera had no effect. Anti-poFcγRII antibody inhibited the enhancement of infection when cells were infected in the presence of anti-PRRSV antibody, but not when cells were infected in the absence of antibody. These results indicate that enhancement of infection in these cells by anti-PRRSV virus antibody is FcγRII-mediated. Identification of the inhibitory FcγR mediating ADE infection should expand our understanding of the mechanisms of pathogenesis for a broad range of infectious diseases and may open many approaches for improvements to the treatment and prevention of such diseases.

## Introduction

Porcine reproductive and respiratory syndrome virus (PRRSV) is an enveloped positive-strand RNA virus in the family *Arteriviridae* [1]. PRRS can cause severe reproductive failure in sows and is associated with the porcine respiratory disease complex in combination with secondary infection [2]–[4]. The virus is present in a majority of swine producing countries around the world and gives rise to significant economic losses in pig farming [5]. Swine are the only known host of PRRSV, and myeloid cells, particularly macrophages and dendritic cells, are the primary permissive cells [6].

Various features of PRRSV infection and the ensuing immune response suggest that PRRSV immunity is aberrant. The acute, viremic infection lasts for 4 to 6 weeks and is followed by a period of persistent viral proliferation in lymphoid tissues that lasts for several months before complete resolution of infection [7]. PRRSV infection can induce significant and specific antibody and B-cell responses to a variety of PRRSV protein [8], [9].

Fcγ receptor (FcγR)-mediated entry of infectious PRRSV immune complexes into macrophages is hypothesized to be a key event in the pathogenesis of the disease [10]–[12]. Infection of alveolar macrophages by PRRSV is significantly enhanced in vitro in the presence of diluted anti-PRRSV antisera [10], and the mean level and duration of viremia are greater in pigs injected with sub-neutralizing antibodies prior to virus challenge than in pigs injected with normal IgG [10], [12]. The prolonged duration of viremia and virus isolation from the tissues of piglets with low maternal antibodies also suggest antibody-dependent enhancement (ADE) of PRRSV [13]. These observations strongly suggest that ADE of PRRSV infection has the potential to enhance the severity of disease and possibly the susceptibility to PRRSV infection in pigs with declining levels of PRRSV-specific antibodies of maternal origin, or with antibodies induced by exposure to wild-type or vaccine strains of PRRSV.

IgG Fc Receptors (FcγRs) comprise a multigene family of integral membrane glycoprotein that exhibit complex activation or inhibitory effects on cell functions after aggregation by complexed IgG. Four different classes of FcγRs, known as FcγRI (CD64), FcγRII (CD32), FcγRIII (CD16) and FcγRIV, have been extensively characterized in mice and humans [14]. Both FcγRI and FcγRIIa have previously been shown to facilitate antibody-mediated dengue virus enhancement in human macrophage [15], [16], and FcγRIIa appeared to be the most effective [17]. FcγRII is a 40-kDa molecule detected on monocytes, neutrophils, eosinophils, platelets and B cells. It has a low affinity for monomeric IgG, preferentially binding multivalent IgG. Porcine FcγRII, previously characterized by our research group, amino acid sequence shows a high similarity to human and mouse FcγRIIb. Since the cytoplasmic domain of this receptor contains a conserved immunoreceptor tyrosine-based inhibitory motif (ITIM), the swine receptor may also have a similar inhibitory function [18]. Therefore, it is important to elucidate the role of porcine FcγRIIb in PRRSV infections in order to better understand PRRSV-porcine cell interactions and the pathogenesis of PRRSV infections.

## Results

### Establishment of stable Marc-145 cell lines expressing poFcγRII (Marc-poFcγRII)

**Figure 1:**
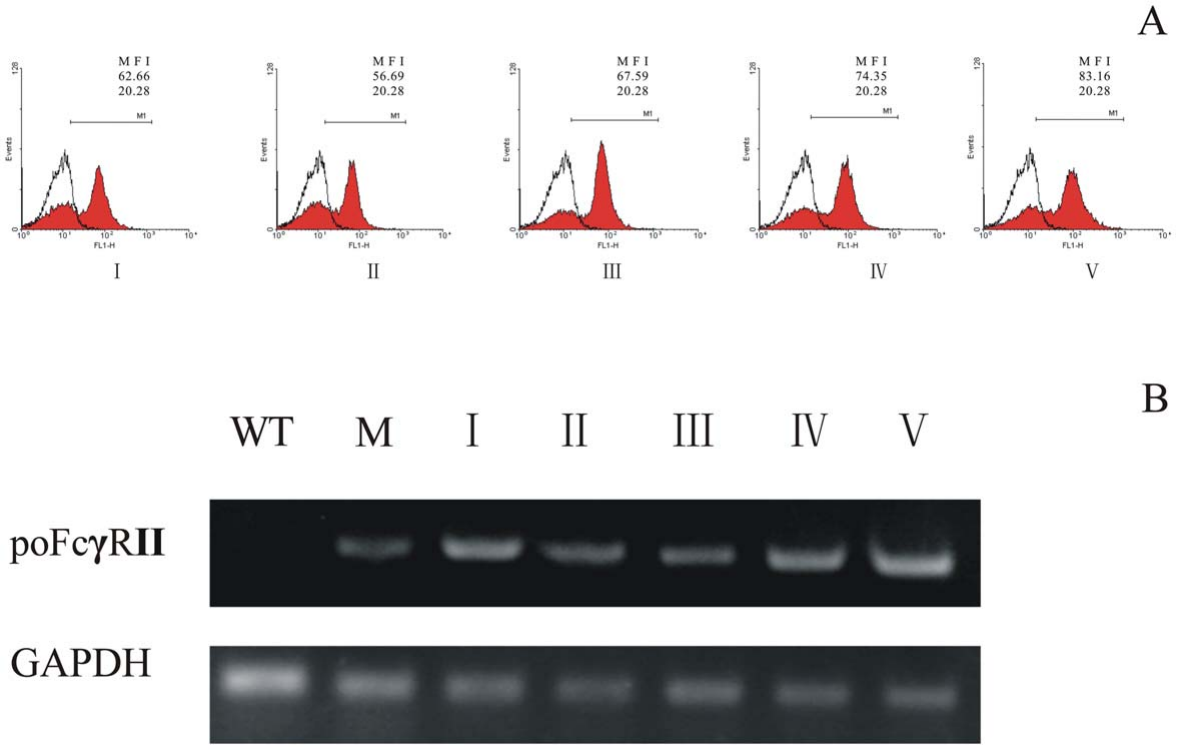
Binding of porcine IgG to poFcγRII on Marc-poFcγRII cells was monitored by flow cytometry. (A). Heat-aggregated IgG was applied to Marc-poFcγRII cells (I–V: passage 1, 5, 10, 15 and 20), followed by washing and incubation with the FITC-conjugated goat anti-porcine IgG. Open graph shows untransfected wild-type Marc-145 cells; Expression of poFcγRII on Marc-poFcγRII cells was determined by RT-PCR (B). Total mRNA was prepared and cDNA was synthesized. This cDNA was then used as a template in polymerase chain reaction (PCR) with poFcγRII or porcine glyceraldehydes 3-phosphate dehydrogenase (GAPDH) specific primers. (lane I–V: passage 1, 5, 10, 15 and 20, lane WT: parent wild-type Marc-145, and lane M: Macrophages).

Marc-145 cell line was selected for transfection with poFcγRII, because it is a permissive cell for PRRSV infection. Three Marc-145 cell lines stably expressing the poFcγRII were established (data not shown), and one of the cell lines, Marc-poFcγRII/1 was selected for further studies. The expression of poFcγRII was verified by flow cytometry and RT-PCR (Fig. 1A and B). Expression of poFcγRII was stable for as long as 20 continuous passages, over a period of 2 months.

### Efficiency of PRRSV replication and progeny virus production in Marc-poFcγRII

**Figure 2:**
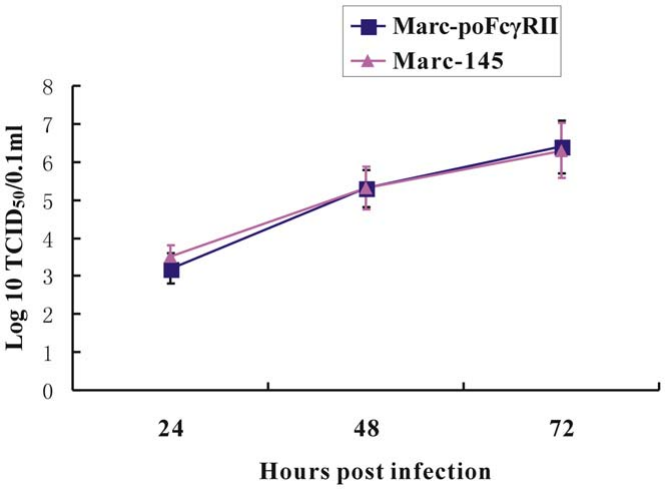
Comparison of PRRSV growth kinetics in Marc-145 and Marc-poFcγRII cells. Marc-145 and Marc-poFcγRII cells were infected with PRRSV BJ-4 strain and harvested at 24, 48 and 72 h post-infection and the virus titers were determined by IFA on Marc-145 cells, respectively. Error bars represented standard errors of mean values from three experiments.

Virus infection and virion production were quantified in the absence of PRRSV antibody using both Marc-poFcγRII and parent Marc-145 cells (Fig. 2). The growth kinetics of PRRSV BJ-4 was similar in the two cell lines. The results indicate that the infection, efficiency of PRRSV replication and mature virion production in Marc-poFcγRII was similar to that of the parent cell line.

### Augmentation of progeny virus production in Marc-poFcγRII cell line by anti-PRRSV antibody

**Figure 3:**
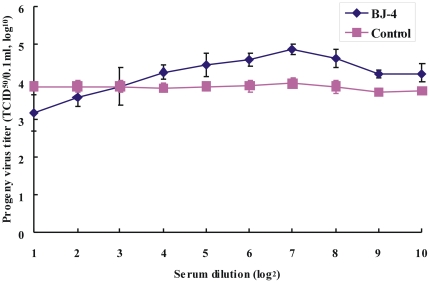
Antibody-dependent enhancement of PRRSV infection revealed by the increase in viral yield as determined by the standard ADE assay. Marc-poFcγRII cells were exposed to PRRSV-antibody complex, prepared by mixing each dilution of the serum sets with an equal volume of DMEM media containing PRRSV BJ-4 strain. Production of infectious progeny PRRSV was quantitated by virus titration. Error bars represented standard errors of mean values from three experiments.

PRRSV-specific antibody were detected by IDEXX commercial ELISA kit and neutralizing antibody test was performed using Marc-145 cells. The sera from one pig with the S/P ratio of 1.2 and the sera neutralizing antibody titer of 1/5.9 were used in the ADE test. Marc-poFcγRII cells were infected with PRRSV BJ-4 strain in the presence or absence of serially diluted anti-PRRSV sera and the virus yield was examined 48 h later. Marc-poFcγRII cells which were infected with PRRSV in the presence of 1/2^4^ to 1/2^10^ dilutions of anti-PRRSV sera produced a significantly higher percentage of virus than those infected in the normal porcine sera (Fig. 3). The anti-PRRSV serum enhanced infection was optimum at a dilution of 1/128. Normal porcine serum, which does not contain anti-PRRSV antibodies did not enhance infection at any dilutions ranging from 1/2 to 1/2^10^. These results suggest that infection with virus-antibody complex results in viral production.

### Analysis of PRRSV-ADE infection in susceptible cells bearing poFcγRII

In order to test whether this enhancement infection specifically applied to poFcγRII positive cells, we pre-incubated PRRSV BJ-4 virions with whole or F(ab')2 fragment of anti-PRRSV IgG at 80 µg/ml and then added the opsonised virions to either Marc-poFcγRII cell lines, porcine alveolar macrophages (PAMs) or Marc-145 cells. Anti-PRRSV IgG enhanced the PRRSV infection in both FcR-bearing cells, Marc-poFcγRII cell line and PAMs (Table 1). No enhancement was seen in the normal Marc-145 cells not bearing FcγRs. Neither the F(ab')2 fragment nor the normal pig IgG had any effect on any cell line (Table 1). Thus, the enhancement of PRRSV infection appears to be due to the presence of poFcγRII.

**Table 1:** Analysis of PRRSV-ADE infection in poFcγRII bearing cell lines.

Group	Progeny virus titer (TCID_50_/0.1 ml log_10_)		
	Anti-PRRSV IgG	Anti-PRRSV F(ab')2	Normal IgG
Marc-poFcγRII	4.61±0.16^A^	3.91±0.13^B^	4.0±0.13^B^
Marc-145	3.97±0.12	3.70±0.05	3.88±0.07
Macrophages	4.79±0.07^A^	3.85±0.05^B^	3.80±0.08^B^

PRRSV BJ-4 virions with whole or F(ab')2 fragment of anti-PRRSV IgG were added to either Marc-poFcγRII cell lines and macrophages or Marc-145 cells. Anti-PRRSV sera enhanced the PRRSV infection in FcR-bearing cells of Marc-poFcγRII cell lines and macrophages infection. Data are shown as mean of three independent experiments. Within the line, means (±standard error) with different superscripts differ significantly (p<0.01).

### Inhibition of anti-PRRSV virus antibody-mediated infection by anti-poFcγRII polyclonal antibodies

**Figure 4:**
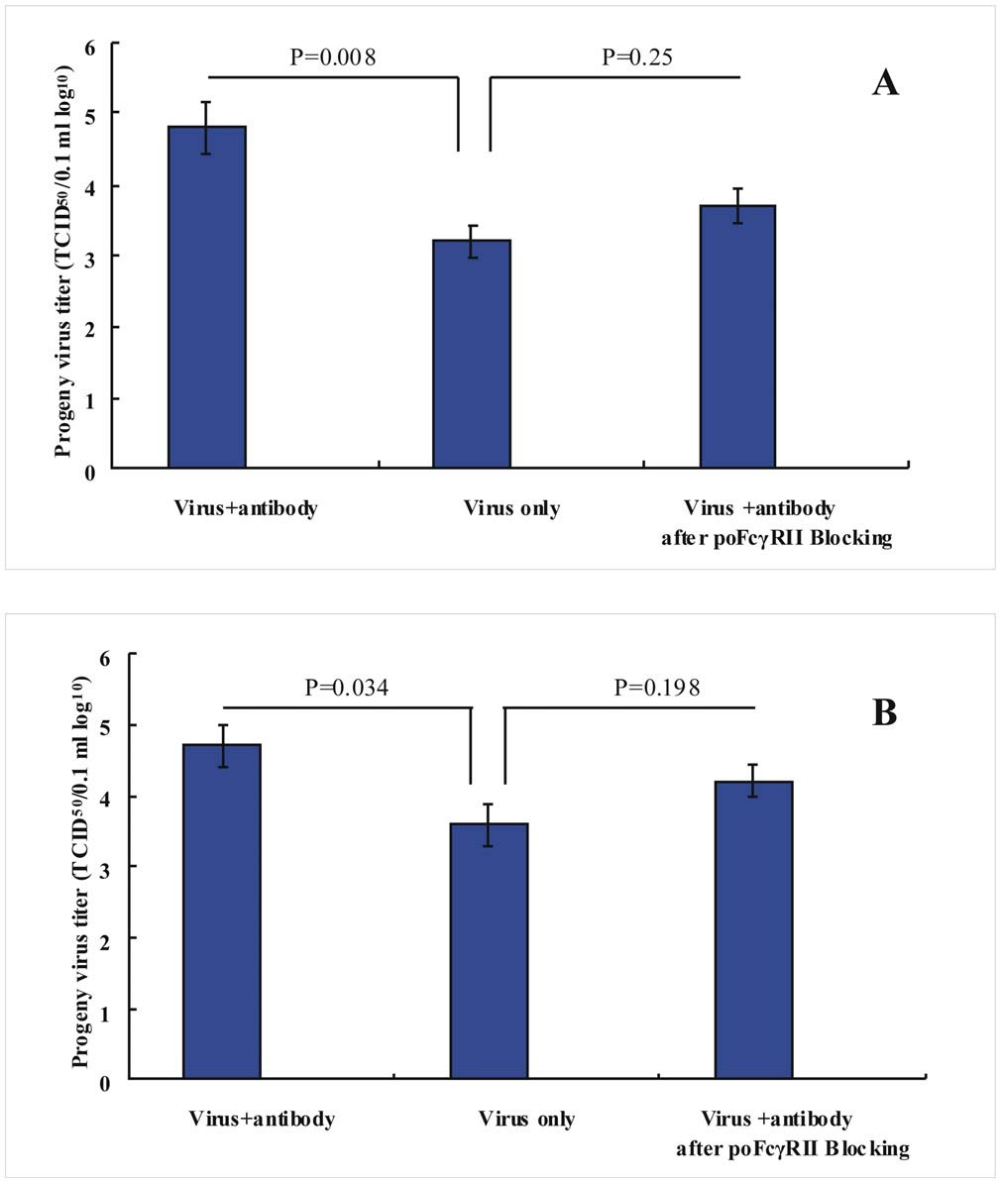
Inhibition of anti-PRRSV virus antibody-mediated infection in Marc-poFcγRII cells (A) and PAMs (B). Cells were incubated with anti-poFcγRII polyclonal antibodies and were then infected with PRRSV in the presence or absence of anti-PRRSV antibody. Production of infectious progeny PRRSV was quantified by virus titration. Error bars represented standard errors of mean values from three experiments.

Rabbit anti-poFcγRII polyclonal antibodies, which are known to inhibit the binding of porcine IgG to poFcγRII, was used to prove that enhancement of PRRSV infection by anti-PRRSV is FcγRII-mediated [19]. The sera were heat inactivated (56°C for 30 min) to remove intrinsic complement activity. Marc-poFcγRII cells and PAMs were incubated with anti-poFcγRII polyclonal antibodies and were then infected with PRRSV in the presence or absence of anti-PRRSV antibody. Anti-poFcγRII antibody inhibited the enhancement of infection when cells were infected in the presence of anti-PRRSV antibody, but not when cells were infected in the absence of antibody (Fig. 4A and B). Control rabbit serum did not inhibit infection in the presence or absence of anti-PRRSV virus antibody (data not shown). These results indicate that the observed enhancement of infection in the presence of anti-PRRSV virus antibody is FcγRII-mediated.

### The infection rates of PRRSV-ADE infection in Marc-poFcγRII cell lines

PRRSV infectivity in Marc-poFcγRII cells under the conditions of ADE infection and control infection was compared at 12, 24 and 48 h post-infection. At 12 h post-infection, there was increased infectivity for ADE-treated cells, but this finding was not statistically significant compared with the control (p = 0.096). As the data shown, a clear and significant increase in infectivity was found after 24 h, with higher numbers of infected cells under ADE infection conditions (Table 2). Therefore the initial PRRSV infectivity was not different between ADE and non-ADE infection in Marc-poFcγRII cell lines, suggesting that post-entry intracellular activities are of greater importance.

**Table 2:** Comparison of percent of PRRSV infectivity in Marc-poFcγRII cells at 12–48 h post-ADE or control infection.

Marc-poFcγRII Treatment	Percent of infectivity (%)		
	12	24	48
PRRSV-ADE	0.30±0.03	3.60±0.15^A^	10.30±0.32^A^
PRRSV-NMS	0.22±0.02	1.23±0.04^B^	3.50±0.20^B^

The infection rates following treatment with PRRSV-specific antibody was determined by immunofluorescence microscopy. Within the same column, mean infectivity (±standard error) with different superscripts differ significantly (p<0.05).

## Discussion

The basis for vaccine development against a wide range of infective agents is the development of protective antibodies. However, the discovery of the phenomena of ADE in the 1960s gave rise to the concern that the development of antibodies could, at times, exacerbate the reaction to a natural infection. Pigs infected with PRRSV develop a strong and rapid humoral response, but the protective effectiveness of these antibodies might be reduced by ADE. The development of significant levels of no-neutralizing antibodies especially early may actually mediate ADE [20].

Antibody-FcγR interaction is known to play a key role in ADE phenomenon. The FcγR-mediated mechanism of ADE in virus infections was first suggested by Halstead et al. who reported that F(ab')2 fragments prepared from IgG did not enhance infection in peripheral blood leukocyte cultures by dengue virus (DV) while whole IgG did [21]. To date, four different classes of FcγRs, known as FcγRI, FcγRII, FcγRIII and FcγRIV, have been recognized in mice. FcγRI (CD64) is a high affinity receptor found mainly on myelomonocytic cells, that can bind to monomeric IgG, whereas FcγRII (CD32) and FcγRIII (CD16) are of lower affinity, binding primarily aggregated IgG or IgG in immune complexes [22], and FcγRIV is a recently identified receptor of intermediate affinity and restricted subclass specificity [23]. Kontny et al. showed that FcγRI mediated ADE of DV infection in U937 cells [15]. In a related study, FcγRIIa was also reported to mediate ADE of DV infection in a human erythroleukemic cell line (K562), which has only FcγRIIa [16]. In the present paper, we have shown that porcine FcγRIIb will mediate ADE of PRRSV infection. The relative viral yield was significantly increased in the presence of sub-neutralizing levels of anti-PRRSV antibody in stable Marc-145 cell lines expressing poFcγRIIb (Marc-poFcγRII) (Fig. 3). The enhancement was not evident for Marc-145 cells not bearing FcγRs. The Fab fragment and the normal porcine sera had no effect (Table 1). Thus, the enhancement of PRRSV load was due to poFcγRII.

Unlike the previously identified FcγRI and FcγRIIa mediating ADE of virus infection, poFcγRIIb is an inhibitory FcγR that can prevent activation of immune cells by recruitment of SHIP (Src Homology 2-containing 5′-inositol phosphatase) to its cytoplasmic ITIM (immunoreceptor tyrosine-based inhibition motif) [18]. During initial studies on ADE it had been assumed that increased virus output resulted from the avid attachment of opsonised virus to FcγRI and FcγIIa receptors, therefore yielding a larger number of infected cells [24], [25]. An alternative proposal was that infection via Fc and FcR ligation also alters the intracellular signaling pathways, resulting in their switching from an anti-viral mode to a viral-facilitating mode. This process has been termed intrinsic antibody-dependent enhancement (iADE) [26], [27]. Infection under conditions of iADE may not only facilitate the process of viral entry into macrophages but also modify the innate and adaptive intracellular antiviral mechanisms. The innate immune response suppression involves a decreased production of reactive nitrogen radicals via nitric oxide synthase (NOS2) suppression and down-regulation of tumor necrosis factor alpha (TNF-α) and IFN-β production through abolished interferon regulatory factor 1 (IRF-1) and NF-κB gene expression, while a marked increase in IL-10 gene transcription and protein production is also observed [28], [29]. In the present work, the infection rate between poFcγRII-mediated ADE infection was compared, and the initial PRRSV infectivity was not different between ADE and non-ADE infection in Marc-poFcγRII cell lines, suggesting that iADE behaviour is of great importance in PRRSV infection (Table 2). The intracellular mechanisms and implications of enhanced pathogenesis of iADE may be the result of the inhibitory signals after interactions of infectious immune complexes with FcγRIIb, the inhibitory Fc receptor. The mechanisms of the inhibitory signal of the FcγRIIb receptor in regulating the viral replication and production need further investigation.

Antibody-dependent enhancement of virus infection is a significant obstacle to the development of effective vaccines for the control of certain human and animal viral diseases. The ADE in PRRSV infection has been suspected as one of the possible reasons for the relative ineffectiveness of vaccination in controlling PRRS. In the present paper we have shown that poFcγRIIb, an inhibitory FcγR can mediate ADE of PRRSV infection, as does FcγRI and FcγRIIa, the activation FcγRs, in the DENV. Identification of the inhibitory FcγR involvement in the ADE process expands our understanding of the mechanisms of pathogenesis for a broad range of infectious diseases and opens approaches for improvement in the treatment and prevention of such diseases. The new ADE assay using Marc-poFcγRII cells is simple and practical, and is useful for defining the role of ADE in the pathogenesis of PRRSV infection.

## Materials and Methods

### Cells, virus and antibodies

Marc-145 cells were used for virus titration and maintained in Dulbecco's modified Eagle's minimal essential medium (Gibco) supplemented with 10% (v/v) fetal bovine serum (FBS, Life Technologies). The PAMs were collected from 4 to 6 week old piglets free of PRRSV, by lung lavage as previously described (Zhang et al., 2006). PAMs were dispensed at 1×10^5^ cells/well into 24-well plate and maintained in RPMI-1640 (Gibco) supplemented with 10% FBS containing an antibiotic-antimycotic mixture of 100 mg/ml streptomycin, 100 IU/ml penicillin and 25 mg/ml amphotericin B (Sigma) and incubated at 37°C in a humidified atmosphere containing 5% CO_2_.

The PRRSV strain BJ-4 was a typical North American (VR2332)-like PRRSV isolated in 1996 in China and its complete genomic sequence has been determined and deposited in GenBank (accession no. AF331831). The virus was supplied by Dr. Hanchun Yang of China Agricultural University. Anti-PRRSV sera were obtained from 3 pigs 50 days following nasal inoculation with 10^4^ TCID_50_ of the PRRSV BJ-4 strain. Control sera were obtained from PRRSV antibody-free pigs of similar age. F(ab')2 fragments were generated by pepsin digestion of isolated IgG and then depleted of undigested antibody and Fc fragments by passage over a protein A-sepharose column. The PRRSV-specific antibody titers were determined by using the commercially available PRRSV antibody detection kit (HerdCheck PRRS; IDEXX) according to the manufacturer's instructions. The virus neutralization (VN) antibody titer was determined in 96-well microtitration plates using Marc-145 cells. Serum samples were heat inactivated at 56°C for 30 min prior to performing the test and serially diluted 2-fold. Each dilution of serum was mixed with an equal volume of PRRSV BJ-4 strain containing 2×10^2^ TCID_50_/ml and incubated for 1 h at 37°C. The serum-virus mixture was transferred to a 96-well plate containing confluent Marc-145 cells, and the cells were analyzed for CPE at 5 days post inoculation. The VN antibody titer was defined as the reciprocal of the highest dilution that inhibited CPE in 50% of the inoculated wells.

The experimental procedure for the collection of porcine alveolar macrophages was authorized and supervised by the Ethical and Animal Welfare Committee of Key Laboratory of Animal Immunology of the Ministry of Agriculture of China.

### Stable expression of poFcγRII on Marc-145 cells

The recombinant eukaryotic cell expression vector of pcDNA3-poFcγRII was constructed as previously described [18]. Marc-145 cells were transfected with *BgI*II-linearized poFcγRII cDNA constructs using Lipofectamine 2000 (Invitrogen) according to the manufacturers' protocols. Transfected cells were selected with 500 µg/ml G418 (Invitrogen) for two weeks and then further selected by the limiting dilution method. The G418-resistant clones were screened by RT-PCR using the PCR protocol described previously [18]. The surface expression of poFcγRII was verified by the binding of porcine aggregated-IgG and assessed by flow cytometry.

### IgG-binding assay

Surface expression of poFcγRII was examined by IgG-binding assay on stably transfected Marc-145 cells as described previously [19]. Porcine IgG was aggregated at 62°C for 20 min. Aggregated IgG was added to the transfected cells. Cells were incubated with the FITC-conjugated goat anti-porcine IgG for 30 min at 4°C, then pelleted and washed twice with PBS. Fluorescent spectra were analyzed by a BD FACSCalibur flow cytometer counting 10,000 cells per sample.

### Infection of cells with PRRSV and PRRSV-antibody complex

Infection of cells with PRRSV-antibody complex was conducted as previously described [10]. BJ-4-specific antibody-positive and antibody-free serums were de-complemented by heat-inactivation at 56°C for 45 min and serially diluted 2-fold from 2^1^ to 2^10^ in DMEM growth media. PRRSV-antibody complex was prepared by mixing each dilution of the serum sets with an equal volume of DMEM media containing 10^4.5^ TCID_50_/ml of PRRSV BJ-4 strain. Virus-antibody mixtures were incubated for 60 min at 37°C. One-tenth of a milliliter of virus-antibody mixture was inoculated in triplicate onto cell monolayers in 24-well cell culture plates. The plates were incubated at 37°C for 60 min. After virus absorption, cells were washed with 1 ml of DMEM and overlaid with maintenance medium with 2% FCS for an additional 48 h at 37°C. At the end of the incubation period, the cells were subjected to 3 cycles of freeze-thawing. The amount of PRRSV in each cell lysate was quantitated by virus titration as described [10]. Virus titers were determined by the method of Reed and Muench and expressed as TCID_50_/0.1 ml.

The infection rates following treatment with PRRSV-specific antibody was determined by immunofluorescence microscopy. Cells infected as above and incubated for 48 h were fixed in cold methanol for 10 min. After washing the cells were incubated for 2 h at 37°C with a monoclonal antibody, 2D6, specific for the nucleocapsid protein of PRRSV (VMRD), followed by the addition of FITC-conjugated sheep anti-mouse IgG antibodies (Sigma) for 1 h at 37°C, after which the cells were counted using a fluorescence microscope. Between antibody incubation and prior to viewing under the microscope, the cells were washed three times with sterile PBS. Ten fields were counted, and mean infectivity (±SE) was calculated.

### Statistical analysis

Data were subjected to one-way analysis of variance (one-way ANOVA). If the P value from the ANOVA was less than or equal to 0.05, pairwise comparisons of the different treatment groups were performed by a least-significant difference test at a rejection level of a P value<0.05.
